# Hypoxic Cell Waves Around Necrotic Cores in Glioblastoma: A Biomathematical Model and Its Therapeutic Implications

**DOI:** 10.1007/s11538-012-9786-1

**Published:** 2012-11-14

**Authors:** Alicia Martínez-González, Gabriel F. Calvo, Luis A. Pérez Romasanta, Víctor M. Pérez-García

**Affiliations:** 1 Departamento de Matemáticas, E. T. S. I. Industriales and Instituto de Matemática Aplicada a la Ciencia y la Ingeniería, Universidad de Castilla-La Mancha, 13071 Ciudad Real, Spain; 2 Departamento de Matemáticas, E. T. S. I. Caminos, Canales y Puertos and Instituto de Matemática Aplicada a la Ciencia y la Ingeniería, Universidad de Castilla-La Mancha, 13071 Ciudad Real, Spain; 3 Servicio de Oncología Radioterápica, Hospital Universitario de Salamanca, 37007 Salamanca, Spain

**Keywords:** Glioblastoma multiforme, Tumor hypoxia, Pseudopalisades, Invasion, Mathematical model

## Abstract

Glioblastoma is a rapidly evolving high-grade astrocytoma that is distinguished pathologically from lower grade gliomas by the presence of necrosis and microvascular hyperplasia. Necrotic areas are typically surrounded by hypercellular regions known as “pseudopalisades” originated by local tumor vessel occlusions that induce collective cellular migration events. This leads to the formation of waves of tumor cells actively migrating away from central hypoxia. We present a mathematical model that incorporates the interplay among two tumor cell phenotypes, a necrotic core and the oxygen distribution. Our simulations reveal the formation of a traveling wave of tumor cells that reproduces the observed histologic patterns of pseudopalisades. Additional simulations of the model equations show that preventing the collapse of tumor microvessels leads to slower glioma invasion, a fact that might be exploited for therapeutic purposes.

## Introduction

Malignant gliomas are the most common and lethal type of primary brain tumor. Survival for patients with glioblastoma multiforme (GBM), the most aggressive WHO grade IV astrocytic glioma (Louis et al. 2007; Wen and Kesari 2008), is about 14 months after diagnosis, using surgery, radiotherapy, and chemotherapy (temozolamide) (Huse and Holland 2010; Van Meir et al. 2010; Clarke et al. 2010; Reardon et al. 2011; Pruitt 2011). GBM periphery typically shows tumor cells infiltrating into the normal brain which often invade the adjacent cortex and the contralateral hemisphere. Indeed, migrating cells which are not eliminated by surgical resection will cause the tumor to recur in the interim of 3–6 months (Onishi et al. 2011; Giese et al. 2003).

**Fig. 1:**
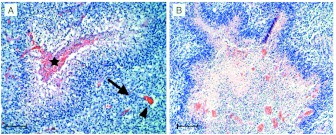
*Pseudopalisades in histopathological samples of GBM*. (**A**) Hypercellular regions appear around centrally occluded vessels (*star*). The standard interpretation (Brat et al. 2004) is that cells migrate away from the degenerated vessels (hypoxic areas) and move toward functional vessels (*arrow*). (**B**) Necrosis formation and the emergence of pseudopalisades is part of a crucial multistep process for the accelerated progression of high grade astrocytic gliomas. The development of an envelope of small scale pseudopalisades may play a relevant role in the global migratory dynamics and invasion of these malignant gliomas driven by vaso-occlusive events occurring at the small cellular scale, leading to the distinctive feature in GBM (Brat et al. 2004). *Scale bars* are 100 and 300 μm, respectively

GBMs are heterogeneous, but are distinguished pathologically from lower grade gliomas by the presence of hypervascularized areas under moderate levels of hypoxia (deficit in oxygenation) and central necrosis usually surrounded by hypercellular regions known as *pseudopalisades* (Gorin et al. 2004). Examples of these hypercellular perinecrotic structures are shown in Fig. 1.

Hypoxia is a feature encountered in most solid tumors (Semenza 2009; Vaupel 2008; Bristow and Hill 2008; Dewhirst et al. 2008). Albeit its incidence and severity varies among patients, it is recognized as a negative clinical prognostic and predictive factor owing to its involvement in various cancer hallmarks (Hanahan and Weinberg 2011; Wilson and Hay 2011) and displays a central role in tumor progression and therapy resistance, especially in GBM (Jensen 2009; Pope et al. 2011). Hypoxia is observed both in the near vicinity of the tumor vasculature (Bristow and Hill 2008; Dewhirst et al. 2008) and at longer distances of about 150 μm from the vessels (Bristow and Hill 2008). Angiogenesis emerges then in response to the proangiogenic growth factors imbalance driven by hypoxic tumor cells such as vascular endothelial growth factor (VEGF) relative to anti-angiogenic growth factors (e.g., angiostatin) (Carmeliet and Jain 2011; Ebos and Kerbel 2011). Although GBM is one of the highest vascularized human tumors, its microcirculation is very inefficient (Jensen 2009) with parenchymal edema and poor maintenance of the blood brain barrier (Jain et al. 2007) leading to a decrease in oxygen and nutrients supply and debris removal. Microvascular hyperplasia, which is a form of angiogenesis morphologically recognized as endothelial proliferation within newly sprouted vessels, is spatially and temporally associated with pseudopalisading necrosis and is believed to be driven by VEGF (Rong et al. 2006).

Experimental studies have hypothesized that pseudopalisade formation could explain the rapid clinical progression of GBM. In fact, it may act as a link among the underlying vascular damage, the development of hypoxia and the hypoxia-induced angiogenesis, leading to necrosis and accelerated outward tumor expansion by a migrating wave of cells. An intriguing observation was that proliferation rates were lower in pseudopalisades than in adjacent astrocytoma for nine different glioma lines (Brat et al. 2004). Since pseudopalisades are mainly composed of hypoxic cells, there exists biological evidence to think that hypoxic cells proliferation indices are lower than normoxic ones and that cell proliferation is not the cause of pseudopalisades formation.

Previous works have proposed pseudopalisade formation as a multistep process (Brat et al. 2004; Rong et al. 2006; Brat and Van Meir 2004): First, tumor cells proliferate and infiltrate through the parenchyma and receive oxygen and nutrients via the intact native blood vessels. Secondly, uncontrolled tumor growth and procoagulant factors expression cause endothelial injury and vascular leakiness resulting in intravascular thrombosis which increase hypoxia in the regions surrounding the vessel (Rahman et al. 2010). Subsequently, tumor cells begin to migrate away from hypoxia, creating a peripherally moving wave that is seen microscopically as pseudopalisading cells, leading to an expansion of the hypoxia zone and central necrosis (see Fig. 1(A)). Meanwhile, hypoxic pseudopalisades tumor cells secrete proangiogenic factors giving rise to more aberrant vessels that will again eventually suffer vaso-occlusions.

**Fig. 2:**
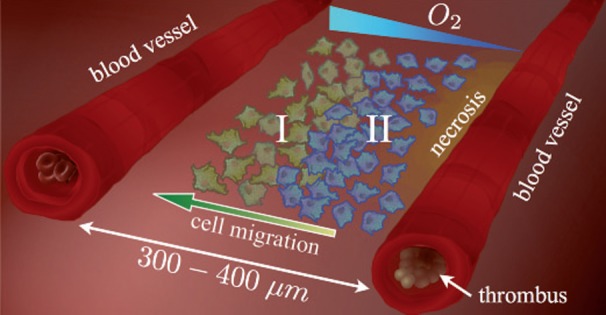
*GBM microenvironment model*. The system modeled here consists of two blood vessels and an evolving embedded population of tumor cells whose phenotype changes according to the oxygen level. Upon thrombosis of one of the blood vessels (the *right* one), due to excessive tumor cell growth and the secretion of thrombotic factors, local oxygen decreases promoting a massive migration of hypoxic glioma cells (labeled as II) toward better oxygenated areas. In the process, hypoxic glioma cells infiltrate among the normoxic glioma cells (labeled as I), which are closer to a functional vessel (the *left* one), creating a transient hypercellularity region (a pseudopalisade). As the pseudopalisading front of glioma cells enlarges around the thrombosed vessel, perivascular necrosis becomes more prominent

In this work, we model the hypercellular regions formation in GBMs perinecrotic areas including the spatiotemporal interplay among normoxic and hypoxic tumor cell, a necrotic core, and the oxygen distribution. Our physio-pathologic scenario is depicted in Fig. 2, and consists of a tumor cell population embedded within two blood vessels arranged in a line domain. Our numerical simulations reveal the formation of a superimposed traveling wave of hypoxic cells that qualitatively reproduces the experimentally observed patterns and provide an estimate of palisade timescale formation, lifetime, and persistence among other prognostic metrics. We also explore the dynamics of tumor spreading under delayed vascular injury and show that somehow, in the framework of our model, preventing vessels from breaking leads to slower tumor invasion speeds that might be explored for therapeutic purposes.

## The Model

### Glioblastoma Compartment Dynamics

GBM is the most heterogeneous primary brain tumor. Studies by Anderson et al. (2009), using three different discrete models, have shown that when low oxygen switch occurs, a large percentage of the populations become growth arrested or removed and the remaining cells are mainly dominated by a single aggressive phenotype. One could include a large range of phenotypes, but such a complex model would involve many unknown parameters.

Actually, a single-cell based model would allow us, in principle, to follow the individual movement of the transformed astrocytes through the brain parenchyma. However, considering that the basic rules behind a model are more important than model details, we discarded both the use of on-lattice models, which are not realistic when cell motion is considered, and off-lattice models, which assume unrealistic cell geometries and/or incorporate unknown cell-cell interactions. Besides, these models often need a large number of unknown parameters and require initial cell configuration which are extremely difficult to validate in in vivo experiments. Thus, since any discrete model will also miss relevant details, we have opted for a continuous model, which does not show any strong spatial localization effects in our simulations.

It is believed that glioma cells follow the migration/proliferation dichotomy (Giese et al. 2003) where highly motile cells exhibit low proliferation rates. The proliferative to invasive switch phenotype cannot be only mutation driven (Onishi et al. 2011; Hatzikirou et al. 2012) and it has been suggested that invasive glioma cells are able to revert to a proliferative program and vice versa, depending on environmental stimulies (Giese et al. 2003; Keunen et al. 2011) such as the oxygen which may drive the transformation. Thus, for each oxygen level, there exists a dominant (fittest) tumor cell phenotype corresponding to certain ratio proliferation/migration rates (Giese et al. 2003). In an *in silico* study, Anderson et al. (2009) studied the evolution of 100 phenotypes with different aggressiveness concluding that the competition between cells induced by oxygenation selects the invasive tumor cell phenotype.

Our modeled system comprises three different compartments: two different coupled tumor cell subpopulations, competing for space and resources (oxygen), corresponding to the two dominant phenotypes, normoxic *C*_*n*_ and hypoxic *C*_*h*_, well described in GBM (DeBerardinis et al. 2007; Giese et al. 2003; Keunen et al. 2011; Onishi et al. 2011). Of the two populations, the first one, *C*_*n*_, consists of normoxic, proliferative, less mobile cells (typically located close to functional blood vessels), whereas the second one, *C*_*h*_, is composed of hypoxic, less proliferative, and more mobile cells. The cell loss is included in a third compartment, *C*_*d*_, of necrotic tissue. The equations governing the interplay among these three densities are as follows: 1a
1b
1c The first terms in Eqs. (1a) and (1b) account for cellular motility. Here, we assume, as in most models (Frieboes et al. 2007; Swanson et al. 2008; Bondiau et al. 2008; Eikenberry and Kuang 2009; Konukoglu et al. 2010; Rockne et al. 2010; Pérez-García et al. 2011) that glioma cell invasion throughout the brain is governed by a standard Fickian diffusion process. Since the hypoxic phenotype is more migratory than the normoxic one (Berens and Giese 1999; Giese et al. 2003; Bristow and Hill 2008; Gorin et al. 2004), hypoxic cell diffusion coefficient *D*_*h*_ is larger than the normoxic one *D*_*n*_.

The second terms in Eqs. (1a) and (1b) employ a classical logistic growth for the tumor cell populations with proliferation times *τ*_*n*_ and *τ*_*h*_, respectively, and a maximum density capacity $C^{\left(M\right)}$. Since normoxic cells are more proliferative than hypoxic ones, then *τ*_*n*_<*τ*_*h*_. Growth is assumed to be space-limited.

**Fig. 3:**
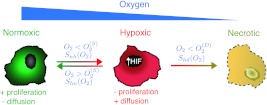
*Oxygenation levels influence tumor cell phenotype*. Oxygenation drives the phenotypic switching mechanisms coupling the populations: Normoxic to hypoxic, *S*_*nh*_, hypoxic to normoxic *S*_*hn*_ and hypoxic to necrotic *S*_*hd*_. High oxygen levels favor the existence of more proliferative phenotypes which are less mobile. On the contrary, cells respond to low oxygen concentrations by expressing less proliferative phenotypes, which are more motile. Finally, hypoxic cells experiencing persistent anoxia eventually die (Brat et al. 2004)

The third and forth terms in Eqs. (1a) and (1b) represent the phenotypic *switching* functions *S*_*ij*_ (see Fig. 3), which are step-like oxygen-dependent functions. Under low oxygenation (below $O_{2}^{(S)}$), normoxic cells change to the hypoxic phenotype (*S*_*nh*_) with a characteristic time *τ*_*nh*_ due to the hypoxia-inducible factor 1*α* (HIF-1*α*) accumulation and the glycolytic and angiogenesis mechanisms initiate (Jewell and Gassmann 2001). Similarly, when oxygenation is sufficient (above $O_{2}^{(S)}$), hypoxic cells recover their oxic phenotype, (*S*_*hn*_) with a characteristic time *τ*_*hn*_. However, hypoxic cells suffering persistent anoxia (below $O_{2}^{(D)}$) start to die (*S*_*hd*_) in Eq. (1c) with a characteristic time *τ*_*hd*_. The parameter Δ*O*_2_ accounts for the characteristic width where the transitions occur around the critical values $O_{2}^{(S)}$ and $O_{2}^{(D)}$.

Glioma cell loss occurs both by a controlled suicide (apoptosis) and by an uncontrolled damage due to extremely unfavorable conditions (necrosis) (Hotchkiss et al. 2009). On the one hand, apoptosis does not correlate with prognosis (Migheli et al. 1994; Schiffer et al. 1995), does not lead to the fill up of space and can be simply incorporated into effective proliferation cell rates in Eqs. (1a) and (1b). On the other hand, necrosis represents a massive cell death and its degree is inversely related to patient survival (Nelson 1983 and Lacroix et al. 2001). Since it happens at a different rate and occupies space, we incorporate *C*_*d*_ into the proliferation limiting terms in Eqs. (1a) and (1b).

### Microenvironment Oxygenation

Though the GBM microenvironment is highly heterogeneous (Bonavia et al. 2011), two of the main chemical agents implicated in its growth are oxygen and nutrients, mainly glucose and lactate (Mendoza et al. 2011; Griguer et al. 2005, 2008; Seyfried et al. 2011). Here, we show that *it suffices* to take oxygen as the key agent driving the collective cell migration dynamics to understand pseudopalisade formation. Moreover, glucose is less scarce than oxygen and can be replaced by other fuels (Beckner et al. 2005; DeBerardinis et al. 2007; Grillon et al. 2011; Mendoza et al. 2011).

Oxygen heterogeneities are very relevant in gliomas and in other tumors (Evans et al. 2004). The spatiotemporal oxygen variation, driving the presence of various phenotypic tumor subpopulations, would help to explain the diversity of responses obtained from the same treatment. Here, a Michaelis–Menten type kinetics is used for the oxygen uptake (Patel et al. 2001; Ferreira et al. 2002) encompassing the feedback by the normoxic and hypoxic cells

2 $$ \frac{\partial O_2}{\partial t} =D_{O_2} \nabla^2 O_2 - \frac{\alpha_n C_n + \alpha_h C_h}{O_2^{\left( T \right)} + O_2}O_2. $$

The first term in Eq. (2) accounts for the oxygen diffusion assuming a homogenous and isotropic diffusion coefficient $D_{O_{2}}$ for simplicity. Although oxygen passes successively through the intracellular fluid, cell membranes and cytoplasm, employing an average diffusion coefficient has been proven to be a good approximation (Tannock 1972; Pogue et al. 2001). The second term models oxygen uptake by normoxic and hypoxic cells at rates *α*_*n*_ and *α*_*h*_, respectively. The saturation Michaelis–Menten constant $O_{2}^{(T)}$ corresponds to the oxygen concentration level at which the reaction rate is halved.

Although it is possible to incorporate the oxygen supply by resorting to extra source functions, here oxygen will be provided initially through the two capillaries located at the tumor boundaries domain and as soon as one of the two blood vessels becomes impaired by a thrombus, the remaining functional one will be the sole source of oxygen (see Fig. 2).

### Parameter Estimation

A large range of cell phenotypes with different parameters exists, but those with better adapted characteristics will be the ones reaching the normoxic region. We resort to mean available experimental values from human glioma models to obtain order-of-magnitude estimates of the intervening parameters in our equations. First, the maximum cell density *C* ^(*M*)^ has been estimated in previous works (see, e.g., Rockne et al. 2010) to be about 10^6^ cell/cm^2^. Oxygen concentration threshold for hypoxic metabolism is cell line dependent but experimental evidence supports for glioma the choice of 7 mmHg (Vaupel 2004). The Michaelis–Menten constant has to be smaller than this parameter yet larger than anoxia threshold, about 0.7 mmHg (Brown and Wilson 2004). We have chosen it to be 2.5 mmHg (Daçu et al. 2003; Wilson 2008).

Oxygen normoxic and hypoxic uptake values are those from U251 glioma cells (Griguer et al. 2008). In Table 1 consumption rates are indicated in Mol/(c s), though they can be put in mmHg/cm by using the ideal gas law.

**Table 1:** Values of the biological parameters used into our model equations

Variable	Description	Value	References
*C* ^(*M*)^	Maximum tumor cell density	10^6^ cell/cm^2^	Rockne et al. (2010)
$O_{2}^{(S)}$	Oxygen concentration switch to hypoxia	7 mmHg	Vaupel (2004)
$O_{2}^{(T)}$	Michaelis Menten oxygen threshold	2.5 mmHg	Daçu et al. (2003)
$O_{2}^{(D)}$	Oxygen level for anoxia	0.7 mmHg	Brown and Wilson (2004)
$O_{2}^{v_{i}},{i=1,2}$	Vessel oxygen concentration	60 mmHg	Wilson (2008) Kimura et al. (1996)
*α*_*n*_	Normoxic cell oxygen consumption	10^−17^ Mol/(c s)	Griguer et al. (2008)
*α*_*h*_	Hypoxic cell oxygen consumption	*α*_*n*_/5 Mol/(c s)	Griguer et al. (2008)
*D*_*n*_	Diffusion coefficient for normoxic cells	6.6⋅10^−12^ cm^2^/s	Tjia and Moghe (2002) Wang et al. (2009)
*D*_*h*_	Diffusion coefficient for hypoxic cells	10 *D*_*n*_ cm^2^/s	Tjia and Moghe (2002) Wang et al. (2009)
$D_{O_{2}}$	Oxygen diffusion coefficient	10^−5^ cm^2^/s	Mueller-Klieser and Sutherland (1984)
*τ*_*nh*_	Phenotype switch time (normoxic to hypoxic)	1 h	Jewell and Gassmann (2001)
*τ*_*hn*_	Phenotype switch time (hypoxic to normoxic)	96 h	Estimated
*τ*_*hd*_	Cell death mean time in anoxia	48 h	Estimated
*τ*_*n*_	Normoxic cell doubling time	24 h	Ke et al. (2000)
*τ*_*h*_	Hypoxic cell doubling time	48 h	Giese et al. (2003) Berens and Giese (1999)
Δ*O*_2_	Sensitivity around transition threshold	0.1 Mol/mm^3^	Estimated
*J*	Oxygen exchange coefficient	0.1 cm	Mazumdar (1992)

Since mean oxygen pressure in arterial blood is around 95 mmHg (Kimura et al. 1996) and venous values are around 30–40 mmHg (Wilson 2008), we will take the oxygen pressure at our boundary capillaries to be $O_{2}^{v_{i}} =60~\mbox{mmHg}$.

The oxygen diffusion coefficient is classically known to be around 10^−5^ cm^2^/s (Mueller-Klieser and Sutherland 1984) while the cell diffusion coefficients are not so readily accessible in vivo. We resort to data from Wang et al. (2009), where the authors assess the invasive capacity of the dominant phenotype linked to the hypoxic one in our model. We will take their median migration rate 29 mm^2^/year as our *fast* diffusion coefficient for hypoxic cells. The used normoxic cell diffusion will be smaller than the hypoxic one, in our case *D*_*n*_=0.1*D*_*h*_.

The hypoxic-normoxic switch time *τ*_*hn*_ can be estimated from Hsieh et al. (2010), where the normalization of the response to hypoxia for U87 glioma cells was between 48 h and 72 h. However, in real situations, *τ*_*hn*_ typically increases with the number of hypoxic cycles experienced by cells until they reach a state of physical balance with HIF1-*α*. This sequence of oxygen deprivation episodes drives genetic alterations in the tumor cells so that HIF1-*α* is stabilized in their nucleus even under oxic conditions, and thus these cells do not return to their previous normoxic state and become irreversibly hypoxic (Semenza 2009). In or model, when *τ*_*hn*_ increases, pseudopalisade formation is enhanced with a larger number of hypoxic cells. This is in accordance with the observation that a greater number of pseudopalisades and hypoxic regions correlates with a higher degree glioma.

Finally, the proliferation parameters can be estimated from typical doubling times that range from 24 to 48 h for glioma cells in vitro for well oxygenated cells, while the hypoxic cells doubling times are assumed to be longer. Table 1 summarizes the typical parameter values employed in our calculations.

### Computational Details

The model given by Eqs. (1a), (1b), (1c) and (2) describes the cell dynamics in the bulk of the tumor under variable oxygenation conditions. As such, it can be studied on any particular geometry either regular or irregular with suitable boundary conditions and/or oxygen sources. It can also be coupled to detailed models of tumor vasculature such as those based on phase-field models (Travasso et al. 2011) and/or combined with oxygen-dependent therapies such as radiotherapy (Jensen 2009; Vaupel 2008; Wilson and Hay 2011). Here, we wish to elucidate the formation of necrotic areas and hypercellular regions such as those observed in gliomas. To do so, we will stick to the simplest possible scenario: one-dimensional tumor sections with blood vessels located on two opposite boundaries and oxygen flow coming from the end points of the domain.

Exchange of gases, nutrients, and waste materials occurs through the thin permeable walls of the vessels, which in our case, are assumed to be capillaries. We will denote the oxygen concentration on the two lateral vessels at a given time *t* by $O_{2}^{v_{1}}(t)$ and $O_{2}^{v_{2}}(t)$. Oxygen flows from vessels to the tissue to balance oxygen pressures. Boundary conditions for the oxygen at the capillaries are

3 $$ \frac{\partial O_2}{\partial x} =\frac{1}{J_i} \bigl(O_2^{v_i}(t) - O_2\bigr), \quad i=1,2. $$

The capillary length in our model simulations is taken to be ≤1 mm to match the real capillary size (Mazumdar 1992). In addition, oxygen concentration in the capillary network does not follow the variations induced by the circulation in the major blood vessels (Daçu et al. 2003). Since our computational domain extends over a small spatial region (vessels distance lower than ≃700 μm), we assume that oxygenation is independent on the position along the capillaries.

Likewise, the oxygen exchange coefficient *J*_*i*_ is considered to be constant and equal for both vessels, therefore, |*J*_1_|=|*J*_2_|=*J*. Bearing in mind that the average blood velocity for capillaries is around 0.1 cm/s (Mazumdar 1992) we can estimate its value to be *J*∼ 0.1 cm. Notice that oxygen spreads either away from or into the vessel depending on oxygen pressures.

It is a fact that GBM only rarely metastatizes out of the brain since GBM cells do not intravasate to the blood vessels (Bellail et al. 2004). Therefore, we impose homogeneous Neumann boundary conditions for the cell densities to supplement model equations (1a), (1b), (1c) and (2).

To solve our model equations we have used second order finite differences in space with an explicit fourth-order Runge–Kutta method in time. Typical parameter values for the spatial step were Δ*x*=Δ*y*=5 μm, and Δ*t*=0.01 s.

In all of the simulations to be presented later, we assume an initial concentration of normoxic tumor cells in the region limited between two functional vessels. Thus, initially, the hypoxic and necrotic cell densities are zero for all *x*∈(0,*L*_*x*_). The initial oxygen distribution is randomized with 24 mmHg as the mean oxygen partial pressure, as described by Vaupel et al. (1989). Except when otherwise stated, we will suppose that the vessel *v*_1_ is disrupted at *t*=0 so that *v*_2_ remains as the only functional vessel through the simulation.

## Results

### Vaso Occlusions Trigger Necrosis, Pseudopalisades, and Vessel-Cooption

We have solved our model equations (1a), (1b), (1c) and (2) by including only the essential cellular biological facts allowing us to understand the palisading necrosis formation, and to get an insight on their formation as well as estimates of their lifetimes and sizes. Once the parameters are chosen in the appropriate realistic ranges (see Table 1), pseudopalisade formation does not require a detailed parameters fine-tuning, but it is a characteristic phenomenon arising in many of our simulations as transient states.

**Fig. 4:**
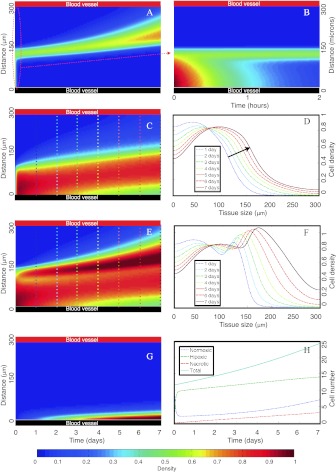
*Spatiotemporal simulations showing the formation of necrotic areas surrounded by hypercellular regions (pseudopalisades)*. Initially, normoxic GBM cells are seeded close to the lower vessel. Pseudo color plots in **A**, **B**, **C**, **E**, and **G** represent normoxic, normoxic (notice the scale), hypoxic, total (hypoxic+normoxic) populations and necrotic tissue densities, respectively. The *horizontal* and *vertical axes* correspond to time in days (except for B in hours) and space in μm, respectively. **D** and **F** depict the normoxic and total (normoxic+hypoxic) cell densities as a function of space for specific times indicated in each curve and also with *vertical color lines* in **C** and **E**. **H** shows the GBM cell number evolution with time for the different populations. At *t*=0, the lower vessel suffers a thrombus, while the upper vessel remains fully functional during the simulation. The parameters used in the simulations are listed in Table 1

In Fig. 4, we present the results for different cell density evolutions when normoxic GBM cells are seeded close to one of two vessels located at the boundaries of a brain tissue slice of length *L*_*x*_=300 μm. The initial (normoxic) tumor cell density surrounds one of the vessels, *x*∈(0,*L*_*x*_/2] (lower black line of Fig. 4), and is zero for *x*∈(*L*_*x*_/2,*L*_*x*_]. We assume well brain oxygenation until the lower vessel is disrupted at *t*=0 so that, at the outset, the hypoxic and necrotic cell densities are zero in the whole computational domain.

After thrombus induction by high adjacent cell density, a normoxic to hypoxic switch, due to oxygen deprivation, leads to an enhanced mobility and reduced proliferation in the perivascular area to find a more nourishing environment to survive (Fig. 4(A)). A complete phenotypic switch occurs in about 1 hour (see Fig. 4(B)) and hypoxic cells (Fig. 4(C)) increase their flux to the upper vessel (in red) which still functional, generating a transient traveling wave of hypoxic cells (see Fig. 4(D) where the arrow shows the movement direction).

Figures 4(E, F) depict the formation of a pseudopalisade in the scale of 1 day that develops during the subsequent days. A morphologic analysis of Figs. 4(E) and F gives about 130 μm as their characteristic width. According to Brat et al. (2004), pseudopalisades with small widths have smaller necrotic cores. In our case, the necrotic core is about 30 μm (Fig. 4(G)) and it is also formed in the same time scale of days. Nevertheless, there is a significant density of migrating hypoxic cells that reach the functional vessel. Actually, the total cell number evolution (Fig. 4(H)) shows that hypoxic cell number increases significantly fast during the first hours after the thrombus but then moderates its growth rate. Normoxic cell numbers initially decay and relapse once the functional vessel has been co-opted. In less than a week, tumor cells invade the total tissue.

**Fig. 5:**
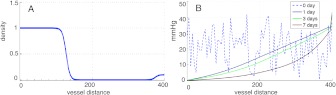
*Initial conditions for the simulation in Fig.* *6*. (**A**) Normoxic cell density. (**B**) Initial oxygen distribution with 24 mmHg as the average oxygen pressure for a healthy brain tissue (Vaupel et al. 1989) and oxygen distribution along the interval of 1, 3, and 7 days after the vessel occlusion in *dark blue*, *green*, and *red lines*, respectively

**Fig. 6:**
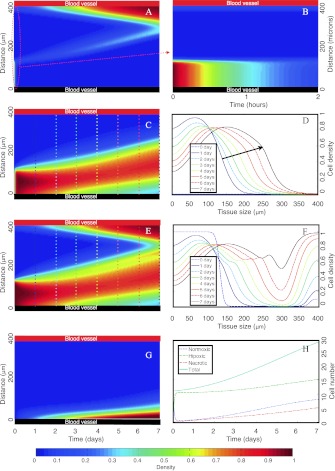
*Spatiotemporal simulations showing the formation of perinecrotic pseudopalisades after a vaso-occlusive event*. In contrast with Fig. 4, here both vessels are initially seeded with normoxic GBM cells. After 7 days, the upper vessel is completely invaded by a high GBM cell density and again a vaso-occlusion event may occur at any moment. All of the subplots and the rest of parameters are as in Fig. 4 and the initial data for oxygen and tumor cells are shown in Fig. 5

In a second set of simulations, displayed in Fig. 6, we study the invasion of a tumor vessel by a pseudopalisading wave of hypoxic cells coming from another impaired vessel. As in the previous case, a high normoxic cell density is seeded around the lower vessel (see Fig. 6), but now we assume that a small number of normoxic tumor cells have already migrated to the upper vessel starting to colonize it (400 μm vessels distance). Initial conditions for the normoxic tumor cell density and oxygenation are shown in Fig. 5.

The simulation starts when the lower vessel collapses induced again by the adjacent high density of proliferating normoxic tumor cells. The upper vessel, fully functional during the simulation, is completely invaded after 7 days by a high tumor cell density that might induce new vaso occlusions (Fig. 6(A)). Hypoxic cells (Fig. 6(C)) generate a transient traveling wave (Fig. 6(D)) where the arrow shows the movement direction. Figures 6(E) and (F) show the pseudopalisade formation in one day that develops during the ensuing 7 days.

A morphologic analysis of Figs. 6(E) and (F) gives about 150 μm as the characteristic width value. The necrotic core is about 60 μm (Fig. 6(G)) and is also formed in the same scale of days. Finally, total cell number evolution observed in Fig. 6(H) is similar to the previous simulation and again normoxic cell numbers have a different growth pattern than the hypoxic one.

It is important to underscore that this minimal model not only reproduces the clinical observations seen in immunohistochemical analysis of histologic sections of GBM samples (Brat et al. 2004), but may also contribute to a better understanding of the origin and evolution of those structures and to find unknown quantities such as the typical palisade lifetime or the relationship between palisade lifetime/size and vessel distance. Moreover, the macroscopic progression of GBM can be partially conceived, from a microscopic point of view, as consisting of a large number of intravascular thrombotic events (caused by unregulated tumor cell proliferation in the vicinity of the vessels) giving rise to the formation and coalescence of small necrotic foci coupled with subsequent episodes of hypoxic cell migrations in search of nearby functional blood vessels, which will eventually suffer new intravascular thrombotic events. The envelope of many of these small-scale pseudopalisades contributes to the high density regions on larger spatial scales as those shown in Fig. 1(B).

### Palisading Waves Invade Faster than the Pure Random Motion Waves

**Fig. 7:**
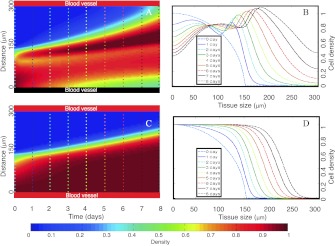
*Palisading waves invade faster than pure random motion waves*. (**A**–**B**) Tumor cell densities evolution along time and space when only the upper vessel is functional while the lower one becomes impaired at *t*=0. Parameters are as in Fig. 4. **A** depicts the total GBM cell density as a function of time, while **B** presents density curves at various times showing the advance of the tumor front. (**C**–**D**) Same as in (**A**–**B**) but when both vessels remain functional. All parameters and initial conditions are taken as in Fig. 4

In principle, it is not clear how vaso-occlusive events relate to the tumor invasion speed. GBM features such as hypercoagulation or abnormal angiogenesis may be linked at the molecular level and hypoxia may coordinate the hypercoagulative activity of GBM cells and (protease-activated receptor) PAR-mediated angiogenic signaling (Svensson et al. 2011). To gain further quantitative insight of vaso-occlusive events influence to the tumor progression speeds, we have performed a series of numerical simulations in Fig. 7.

Subplots A, B correspond to the case discussed previously where the upper vessel works normally but the lower becomes impaired due to the thrombus formation thrombus. On the contrary, subplots C,D show the tumor progression when both vessels remain functional. In the first case, the functional vessel is invaded by the tumor after 8 days by a high tumor cell density (more than 45 %). However, when no thrombus is formed and both vessels stay functional (subplots C and D) invasion becomes significantly slower; requiring 13.5 days to attain the same tumor cell density around the upper vessel.

Figure 7 displays the coexistence of two mechanisms driving invasion: one purely diffusive and similar to that governed by a free growth (when resources are not limited) obeying the Fisher–Kolmogorov equation, as described by Swanson et al. (2008) and related works. The second one shows an accelerated invasion caused by micro thrombi formations in the tumor vessels. Thus, on the basis of our model, it appears that vaso-occlusive events in GBM may play a key role in accelerating the tumor invasion process through pseudopalisade formation. This suggests that the use of chemical agents slowing-down the vessel impair might delay tumor progression in GBM patients. This fact will be discussed thoroughly in Sect. 4.

### Vessel Distance, Palisade Lifetime and Necrotic Width Relationship

**Fig. 8:**
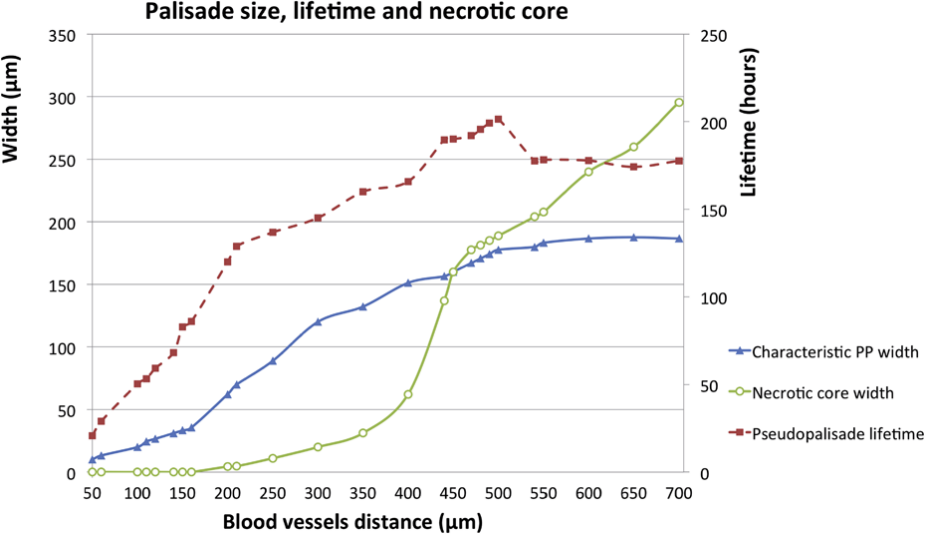
*Occluded-functional vessel distance, palisade width and its lifetime link*. In all simulations, GBM normoxic cells are seeded close to the impaired vessel with a small fraction of them already invading the functional vessel. Initial conditions are chosen similar to those of Fig. 6 and proportional to the tissue size. At *t*=0, one of the vessels collapses while the second remains functional during the simulated time window. Characteristic pseudopalisade width and lifetime data are labelled by *blue triangles*, and *red squares*, respectively. Also shown is the dependence of the necrotic core width with the tissue size (*green circles*). The horizontal axis represents the blood vessels distance (tissue size) in μm. *Vertical axes* label the palisade and the necrotic foci widths in μm (*left* one); pseudopalisade lifetime in hours (*right* one). The parameters used in the simulations are listed in Table 1

To further substantiate our results, we have carried out a numerical study of the relation between the separation between a thrombotic vessel and another functional vessel, the palisade width and its lifetime, as depicted in Fig. 8. The characteristic width of the pseudopalisade corresponds to the dimensions of the hypercellular region and its lifetime is the time elapsed since the palisade is formed until it disappears. Although the standard distances between blood vessels are in the range of 100 to 400 μm, due to the irregular tumor vasculature, we have considered a wider range (from 50 to 700 μm) assuming that those histologic samples showing distances larger than 700 μm will have other closer fonts not visible in pathological analyses due to its spatial arrangement.

In our computational window, the blood vessels distance equals the tissue size and so when this is smaller than 100 μm the formation of pseudopalisades is practically nonexistent. Figure 8 shows how the pseudopalisade size depends on vessels distance (it may be also expected to be roughly proportional to the vessel size), growing in a linear fashion as the distance increases until it reaches a plateau value close to 200 μm.

Figure 8 shows that if the distance from a thrombotic vessel to the nearest functional vessel is short enough, the cells arrive to better oxygenated areas without experiencing severe hypoxic stresses (thus diminishing their death) and reducing the palisade lifetime. However, if this distance is larger than 150–200 μm (or if the broken vessels are larger), there will be a considerable number of hypoxic cells unable to escape from severe hypoxia giving rise to larger necrotic areas and longer palisade lifetimes. The model, although biologically simple and solved on a line domain, supports the hypothesis that large pseudopalisades are more persistent. This theory is well based on the pathology fact that pseudopalisades surrounding larger broken vessels are easier to observe (cf. Fig. 8).

## Impact and Therapeutic Implications

### Impact

It is well known in biological contexts that some aspects of glioma dynamics can be understood in terms of two cell subpopulations corresponding to two dominant phenotypes (Giese et al. 2003; DeBerardinis et al. 2007; Keunen et al. 2011; Onishi et al. 2011; Hatzikirou et al. 2012). This means that minimal mathematical models such as those based on Fisher–Kolmogorov type equations accounting for GBM progression (Frieboes et al. 2007; Swanson et al. 2008; Bondiau et al. 2008; Eikenberry and Kuang 2009; Konukoglu et al. 2010; Rockne et al. 2010; Pérez-García et al. 2011) may benefit from incorporating the two different phenotypes and necrosis as it is done in this paper. On the other hand, the development of an envelope of small scale pseudopalisades may play a relevant role in the global migratory dynamics and invasion of these malignant gliomas driven by vaso-occlusive events occurring at the small cellular scale, leading to the distinctive GBM feature of showing significant necrotic areas, as observed in clinical imaging such as the on exhibited in Fig. 1(B).

However, in order to incorporate necrosis not only phenomenologically, as in Pérez-García et al. (2011), it is necessary to take into account the basic intervening steps involved in palisade formation discussed in Brat et al. (2004) and Brat and Van Meir (2004). Our simulations show that vascular damage is not only a key event in the necrosis formation but also a driving force for migration which is linked to the cell densities evolution.

Other possible extensions regard the presence of a small number of treatment-resistance (cancer-stem) cells in GBM and whether their metabolism can be effectively targeted. Indeed, determining whether this small fraction mainly depends on aerobic glycolysis or on oxidative phosphorylation is relevant (Pistollato et al. 2010). It is known that in glycolytic tumors as GBM, oxidative phosphorylation is not completely shut down since it provides ATP even under low glucose demand (Vander Heiden et al. 2009; Mathupala et al. 2010).

While our distinction of the tumor cell phenotypes has relied on their oxic state, we anticipate that, among the migratory hypoxic cells seen in pseudopalisades, a small fraction of them is formed by treatment-resistance cells. Actually, our simulations suggest that during the time window where hypoxic cells migrate any oxygen dependent treatment will exhibit a poor clinical response (e.g., radiotherapy). Oxygenation importance is further supported by recent experiments by Vlashi et al. (2011) in which it was observed that in various GBM cell lines, progenitor cells, and those exhibiting a stem-cell-like behavior consume less glucose and produce less lactate while maintaining higher ATP levels than their differentiated progeny. They concluded that GBM stem cells were mostly oxydative. Even from an evolutionary point of view, the collective migration of hypoxic GBM cells when forming pseudopalisades appears to be a selection mechanism of the fittest cell phenotypes.

### Therapeutic Implications

Our result that *targeting vaso-occlusions might delay GBM tumor progression* has potential therapeutic implications, since increasing the current GBM modest survival would constitute a big success due to its poor prognosis.

Thromboembolism, (large vessel occlusion), is well recognized as a major cancer complication and known to be a common cause of death in cancer patients, however, the contribution of chemotherapeutics, tumor cells, endothelium, and procoagulants in promoting thrombus continues to be investigated (Khorana and Francis 2008; Furie and Furie 2006; Zwicker et al. 2007; Green and Kwaan 2009). Specifically, gliomas have 25 %–30 % of patients sustaining venous thromboembolism (VTE) (Streiff et al. 2004; Simanek et al. 2007) and it is known that glioma cells secrete pro-thrombotic factors (Bastida et al. 1984) and that the more tumoral tissue is removed the less-likely are the patients to die from VTE (Brose and Lee 2008; Simanek et al. 2007). This fact has led to the consideration of thromboprophylaxis for glioma patients with higher potential risks of VTE (Hamilton et al. 1994; Batchelor and Byrne 2006; Jenkins et al. 2010; Khorana and Francis 2008).

In addition to the VTE prevention there are several direct mechanisms of action of some anticoagulants, such as low molecular weigh heparine (LMWH), on tumor cells: direct cell killing (Dos Santos et al. 2010), anti-angiogenic effects (Svensson et al. 2011) and others (see, e.g., Green and Kwaan 2009, Chap. 15). Our model results seem to imply that an additional indirect antitumoral effect from thromboprophylaxis might be expected related to the tumor invasion delay, different from the direct antitumoral effect or the VTE prevention. The main limitation in using LMWH in post-operative patients is the bleeding risk, however, a limited phase II clinical study of LMWH for high grade glioma patients by Robins et al. (2008) has shown that it is safe. A phase III trial with a small number of patients seems to confirm this trend (Perry et al. 2010).

If, as predicted, preventing capillar thrombosis induced by the tumor, results in a growth tumor delay, a synergistic positive effect with radiation therapy is expected. Reducing hypoxia would lead to an enhanced tumor radio-sensitivity, that might provide an extra benefit in survival.

Whether these predictions are clinically relevant remains to be studied, testing in animal models if LMWH induces a significant reduction of palisade formation delaying tumor progression and then in clinical trials. Mathematical models might contribute to design therapies and we expect our work to stimulate theoretical and experimental studies to follow in this direction.

## Conclusions

In this work, we have put forward a simple continuous mathematical model allowing for the description of the formation of hypoxic hypercellular regions around necrotic cores in glioblastoma multiform: the so-called pseudopalisades. Pseudopalisading necrosis and microvascular hyperplasia are two of the most powerful predictors of poor prognosis among invasive gliomas. These structures could contribute to accelerate growth properties that characterize the low grade to high grade astrocytic glioma transition.

Our model quantifies the migration process influenced by the cell phenotypic switch under hypoxic conditions caused by vaso-occlusive episodes. The model evidences that it is possible to observe the dependence between thrombotic-functional vessel separation, palisade width, lifetime, and its necrotic core. We have also provided quantitative metrics of the characteristic necrotic core, times of formation, lifetimes and width of pseudopalisades after a vaso-occlusion. We stress that these important metrics are very challenging to measure in vivo because of the small spatial scale of the phenomena involved and the fact that biopsies do not provide dynamical information in time, but only a fixed. In addition, our results indicate that smaller pseudopalisades would show shorter lifetimes and reduced necrotic cores while larger pseudopalisades would display longer lifetimes until reaching a threshold around the eighth day after the vaso-occlusion. From our calculations, pseudopalisades tend to present a characteristic width of 200 μm when blood vessel distances are larger than 500 μm (within the range explored up to 700 μm).

Furthermore, our simulations show that palisading waves lead to a faster invasion than that by a pure random motion invasion with unlimited resources, implying that the mechanism of vessel break up might accelerate the glioblastoma progression. This suggests that targeting vaso occlusion events, probably by anticoagulants such as low molecular weight heparine, might be of potential interest for therapeutics aimed at delaying glioblastoma growth.
